# Perioperative Nivolumab and Ipilimumab with Chemotherapy and Chemoradiation for Resectable Gastric and Gastroesophageal Junction Adenocarcinoma: A Phase 1/2 Non-Randomized Clinical Trial

**DOI:** 10.3390/cancers18142198

**Published:** 2026-07-08

**Authors:** Mariela A. Blum Murphy, Lianchun Xiao, Matheus Sewastjanow-Silva, Xumei Wang, Brian D. Badgwell, Paul F. Mansfield, Naruhiko Ikoma, Cindy M. Pabon, Jeffrey H. Lee, Manoop S. Bhutani, Brian Weston, Emmanuel Coronel, Grace L. Smith, Emma B. Holliday, Jessie Tian, Anas M. Barabrah, Prajnan Das, Bruce D. Minsky, Rebecca E. Waters, Jeannelyn S. Estrella, Jenny J. Li, Jaffer A. Ajani

**Affiliations:** 1 Departments of Gastrointestinal Medical Oncology, The University of Texas MD Anderson Cancer Center, 1515 Holcombe Blvd, Houston, TX 77030, USA; 2 Departments of Biostatistics, The University of Texas MD Anderson Cancer Center, 1515 Holcombe Blvd, Houston, TX 77030, USA; 3 Departments of Surgical Oncology, The University of Texas MD Anderson Cancer Center, 1515 Holcombe Blvd, Houston, TX 77030, USA; 4 Departments of Gastroenterology, Hepatology and Nutrition, The University of Texas MD Anderson Cancer Center, 1515 Holcombe Blvd, Houston, TX 77030, USA; 5 Departments of Gastrointestinal Radiation Oncology, The University of Texas MD Anderson Cancer Center, 1515 Holcombe Blvd, Houston, TX 77030, USA; 6 Departments of Anatomical Pathology, The University of Texas MD Anderson Cancer Center, 1515 Holcombe Blvd, Houston, TX 77030, USA

**Keywords:** gastric cancer, immunotherapy, neoadjuvant therapy, pathologic complete response, nivolumab

## Abstract

Background/Objectives: Immunotherapy (IO) has demonstrated survival benefits in metastatic gastroesophageal cancers, and current data supports perioperative IO in localized adenocarcinomas. Radiation may further enhance IO response through immunologic priming. This study evaluates the feasibility, safety, and preliminary efficacy of incorporating IO into a chemoradiation-based perioperative strategy for resectable gastric and gastroesophageal junction (GEJ) adenocarcinoma. Methods: This single-arm, phase I/II study enrolled adults with untreated, locally advanced, resectable gastric or GEJ adenocarcinoma between February 2019 and June 2023. The treatment protocol consisted of induction chemotherapy (oxaliplatin + 5-fluorouracil), induction IO (nivolumab + ipilimumab), concurrent immune-chemoradiation (nivolumab, 5-fluorouracil, and 45 Gy IMRT/VMAT), surgical resection, and adjuvant nivolumab for residual disease. Primary endpoints were safety and feasibility; secondary endpoints included the pathologic complete response (pCR), R0 resection rate, disease-free survival (DFS), overall survival (OS), and biomarker analysis. Results: In total, 30 patients were enrolled, and 23 underwent resection. Grade 4 treatment-related toxicities occurred in three patients (10%), including acute kidney injury, myocarditis/myositis/myasthenia gravis overlap syndrome, and neutropenia. Among surgical patients, the pCR rate was 39.1% (95% CI: 19.7–61.5%), and the intention-to-treat pCR rate was 30% (95% CI: 14.7–49.4%). R0 resection was achieved in 87% of cases. Median DFS among resected patients was 40.2 months (95% CI: 21.6–NE). Median OS was 43.7 months (95% CI: 30.7–NE), with 2-, 3-, and 5-year OS rates of 73.3%, 57.5%, and 47.9%, respectively. Conclusions: This multimodality approach incorporating IO with chemotherapy and chemoradiation demonstrated a manageable safety profile and an encouraging pCR rate, supporting further evaluation.

## Simple Summary

Patients with localized gastric and gastroesophageal junction cancers often require chemotherapy both before and after surgery. Recent advances in immunotherapy have improved survival outcomes, and studies in advanced disease suggest that combining immunotherapy with chemotherapy and radiation may further enhance the immune response against cancer. In this study, we evaluated the safety and effectiveness of adding immunotherapy to an intensive treatment approach that included chemotherapy, radiation, and surgery for patients with localized gastric and gastroesophageal junction adenocarcinomas. We also assessed the tumor response and the duration of disease-free survival following treatment. Our findings showed that this combined treatment strategy was manageable for most patients and resulted in a higher rate of complete tumor eradication at the time of surgery. These results suggest that incorporating immunotherapy into standard treatment may improve patient outcomes and support further investigation in larger clinical studies.

## 1. Introduction

Despite a steady decline in the incidence of gastric cancer (GC) over recent decades, its management remains a major global challenge. GC accounts for approximately 7.7% of all cancer-related deaths and stands as the fourth-leading cause of cancer mortality worldwide [1,2]. In the United States, data from the Surveillance, Epidemiology, and End Results (SEER) program indicate that only about 32% of patients present with localized disease [3], for whom curative-intent surgery is a potential option.

Accurate staging is essential for determining optimal management [4]. In localized GC, treatment strategies vary globally. In the United States, standard approaches include postoperative chemoradiation or perioperative chemotherapy with the FLOT regimen (5-fluorouracil, oxaliplatin, docetaxel) ± durvalumab [5,6], whereas Asian approaches commonly use adjuvant S-1/docetaxel or capecitabine-oxaliplatin [7,8].

The introduction of immunotherapy (IO) has transformed the treatment landscape of metastatic gastroesophageal cancers, with multiple studies demonstrating survival benefits when IO is combined with chemotherapy [9,10,11,12,13]. Chemotherapy agents such as oxaliplatin may further enhance IO efficacy by promoting an immunogenic tumor microenvironment and enhancing T-cell infiltration [14]. The Checkmate 577 trial further supported the role of IO by showing improved disease-free survival (DFS) with adjuvant nivolumab in patients with esophageal or gastroesophageal junction (GEJ) cancers who had residual disease following neoadjuvant chemoradiotherapy and surgery [15]. Radiation itself also enhances tumor immunogenicity through immunogenic cell death, antigen release, dendritic cell activation, and T-cell priming, providing a strong rationale for combining IO with chemoradiation [16].

Based on the established activity of immune checkpoint inhibitors in advanced gastroesophageal cancers and the potential for radiation-induce immune priming, we hypothesized that integrating IO into our institutional chemotherapy and chemoradiation platform could enhance tumor regression while maintaining acceptable safety. Therefore, we conducted a single-arm phase I/II study evaluating perioperative nivolumab and ipilimumab combined with chemotherapy, chemoradiation, surgery, and adjuvant nivolumab in patients with resectable gastric and gastroesophageal junction adenocarcinoma. Primary objectives were safety and feasibility, with secondary objectives including the pathologic complete response (pCR), R0 resection rate, DFS, and biomarker analysis at predefined treatment intervals.

## 2. Materials and Methods

### 2.1. Study Design and Participants

**Figure 1:**
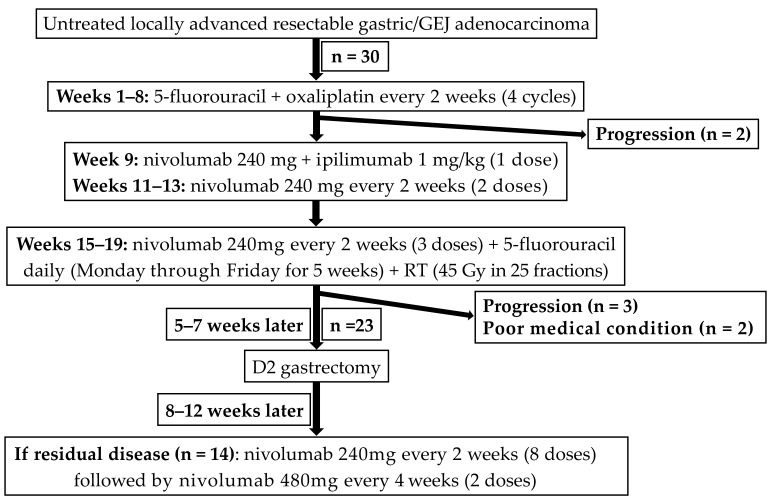
Study diagram.

This was a non-randomized, single-arm phase I/II study conducted at The University of Texas MD Anderson Cancer Center in Houston, Texas. Eligible patients were aged ≥18 years, with previously untreated, locally advanced, resectable gastric or GEJ adenocarcinoma (Figure 1). Comprehensive staging including cross-sectional imaging, endoscopic ultrasound and diagnostic laparoscopy with negative peritoneal cytology was required to confirm resectability. Additional eligibility criteria included an Eastern Cooperative Oncology Group performance status (ECOG-PS) of 0–1, adequate organ function, and no contraindications to the planned multimodality treatment. Participants of reproductive potential were required to use effective contraception throughout this study and for 5–7 months following completion of adjuvant therapy. All enrolled patients agreed to provide tumor tissue samples for further assessment. Microsatellite status and programmed death-ligand 1 (PD-L1) expression were obtained but not used to determine eligibility.

Patients with active autoimmune disease were excluded, with the exception of well-controlled type I diabetes, hypothyroidism requiring only hormone supplementation, and autoimmune skin disorders not requiring systemic therapy. Individuals requiring prolonged corticosteroid or other immunosuppressive therapies for active disease(s) were ineligible. Additional exclusion criteria include known human immunodeficiency virus (HIV) infection, hepatitis B virus (HBV), or untreated hepatitis C virus (HCV). Patients with a prior malignancy within the preceding years were excluded unless the condition was considered locally curable (e.g., squamous cell skin cancer, carcinoma in situ of the prostate, cervix, or breast). Prior exposure to checkpoint inhibitors or other immune-based therapies targeting T-cell co-stimulatory pathways also precluded enrollment.

The study protocol and all amendments were approved by the institutional review board. This study was conducted in accordance with the Declaration of Helsinki (as revised in 2013), and all patients provided written informed consent.

### 2.2. Treatment Protocol

Step 1: Induction Chemotherapy (Weeks 1–8)

Patients received induction chemotherapy in 14-day cycles (weeks 1, 3, 5, and 7) for a maximum of 4 cycles. Treatment included the following:

- Oxaliplatin 85 mg/m^2^ administered intravenously (IV) over 2 h on Day 1.
- 5-fluorouracil 2.4 g/m^2^ delivered as a continuous 48 h intravenous infusion beginning on Day 1.

Step 2: Immunotherapy (Weeks 9–14)

Following induction chemotherapy, patients proceeded with immunotherapy:

- Nivolumab 240 mg IV infused over 30 min on Day 1 every 2 weeks for a total of three doses (Weeks 9, 11, and 13).
- Ipilimumab 1 mg/kg IV infused over 30 min, administered once on Day 1 of Week 9 concurrently with nivolumab.

Step 3: Concurrent Immuno-Chemoradiation (Weeks 15–19)

Patients then received five weeks of concurrent immunotherapy, chemotherapy, and radiation:

- Nivolumab 240 mg IV every 2 weeks for three doses (Weeks 15, 17, and 19).
- 5-fluorouracil 250 mg/m^2^ IV daily, Monday through Friday, for a total of 25 treatments days across Weeks 15–19.
- Radiation therapy with a total dose of 45 Gray (Gy) delivered in 25 fractions using intensity-modulated radiation therapy (IMRT) or volumetric modulated arc therapy (VMAT). Three-dimensional conformal radiation therapy was allowed at the discretion of the treating physician. Radiation fields were defined according to pretreatment imaging, endoscopic findings and involved nodal basins. The clinical treatment volume (CTV) included the gross tumor volume (GTV), 3 cm mucosal margin around the GTV, involved nodes, and elective nodal regions at risk, all expanded by 1 cm in all directions. The planning target volume (PTV) included the CTV expanded by 0.5 cm in all directions.

Step 4: Surgical Resection

Surgery was scheduled approximately 5–7 weeks after completion of radiation therapy (RT). The surgical approach included either partial or total gastrectomy and D2 lymphadenectomy based on tumor location and multidisciplinary surgical assessment.

Step 5: Adjuvant Immunotherapy

Patients with residual disease on surgical pathology specimens, irrespective of PDL-1 status, received adjuvant nivolumab:

- Nivolumab 240 mg IV every 2 weeks for 8 doses (approximately 16 weeks) followed by Nivolumab 480 mg IV every 4 weeks for 2 additional doses, beginning 2 weeks after completion of the initial eight infusions.

Adjuvant IO commenced 8–12 weeks after surgery and continued for a total of approximately 6 months.

### 2.3. Nutritional Support Considerations

Patients presenting with significant impairment in oral intake (<600 Kcal/day, limited to liquids) were advised to undergo placement of a feeding jejunostomy tube, either laparoscopically or percutaneously. Nutritional support and hydration were closely monitored throughout the treatment, with heightened attention during Step 3, when radiation-related toxicities may further impair oral intake. If enteral access was not required before surgery, a feeding jejunostomy was placed at the time of surgery (Step 4) to support postoperative nutritional needs.

### 2.4. Outcomes

Following induction chemotherapy, and prior to immuno-chemoradiation, patients underwent endoscopy for tissue banking and biomarker assessment. After neoadjuvant therapy completion, clinical response was evaluated by endoscopic biopsy and cross-sectional imaging.

Definitive treatment response was determined by histopathologic evaluation of the surgical specimen. Pathologic response was categorized into three groups:

1. Pathologic complete response (pCR): no residual carcinoma detected either in primary tumor or in any lymph node.
2. Partial response: <50% of residual viable tumor cells.
3. Limited response: ≥50% of residual viable tumor cells in the specimen.

All patients who received at least one cycle of chemotherapy were considered for safety evaluations, which included documentation of treatment-related deaths, premature treatment discontinuation due to toxicity, adverse events (AEs), and serious adverse events (SAEs), graded according to NCI-CTCAE version 5.0. Patients were monitored for a minimum of 100 days after the final dose of study drug, and followed until AEs were resolved, returned to baseline, or were deemed irreversible.

Long term survival follow-up was planned for up to three years following completion of therapy for the last patient enrolled.

### 2.5. Statistical Analysis

The primary objective of this study was to evaluate the safety and toxicity profile of IV nivolumab in combination with ipilimumab following induction chemotherapy and subsequently with fluoropyrimidine-based RT, in patients with localized GEJ and/or gastric adenocarcinoma. Secondary objectives included assessment of treatment efficacy, estimation of DFS, and evaluation of changes in tumor stroma composition before and after immunotherapy and radiation.

Toxicity monitoring followed the Bayesian method of Thall, Simon, and Estey [17]. The trial incorporated a predefined early stopping rule: enrollment would be halted early if Prob (*p* > 0.30 data) > 0.85, where *p* denotes the probability of treatment-related toxicity. This rule was evaluated in cohorts of six patients. Assuming a beta (0.6, 1.4) priori for *p*, early termination would occur if, among the first 12 patients enrolled, six or more patients experience treatment-related toxicities. If the study proceeded without early termination, a sample size of 30 evaluable patients was estimated to yield a Bayesian 90% posterior credible interval of approximately 0.176–0.438, under a true toxicity rate of 30%.

Continuous variables were summarized using descriptive statistics (e.g., median and range), and categorical variables using frequency and percentages. The pCR rate was estimated along with a corresponding exact 95% confidence interval (CI).

Time-to-events outcomes, including overall survival (OS) and DFS, were analyzed using the Kaplan–Meier method.

- OS was defined as the time from diagnosis to death or last follow-up (for patients alive).
- DFS was defined as the time from surgery to recurrence/progression, death or last follow-up, whichever occurred first.

Safety data were summarized separately for the neoadjuvant and adjuvant phases by organ system, grade, and attribution. Preliminary efficacy measures, including clinical and pathological response rates, were estimated with exact 95% CI. Changes in tumor stroma composition and tumor biomarkers before and after treatment were summarized using descriptive statistics and evaluated statistically using the Wilcoxon signed rank test.

A *p*-value of less than 0.05 was considered statistically significant in this study. All statistical analyses were conducted using IBM (Armonk, NY, USA) SPSS Statistics v29.

### 2.6. Role of Funding Source

This was an investigator-initiated trial (CA209-9KL) supported by Bristol Myers Squibb (BMS) (Princeton, NJ, USA). The sponsor had no role in study design, data collection, analysis, interpretation, or manuscript preparation.

## 3. Results

From February 2019 to June 2023, 36 patients were enrolled. Six patients were ineligible or pursued alternative treatment strategies, resulting in a final evaluable cohort of 30 patients. Baseline characteristics are outlined in Table 1. The median age was 58 years old, with a balanced cohort of male (57%) and female (43%) participants. Most participants were Caucasian (73%), and not Hispanic or Latino (67%). Most tumors were centered in the stomach (77%) rather than the GEJ (23%), and most patients had at least stage III disease (60%) with diffuse (63%) and poorly differentiated (67%) histology. Among patients with available microsatellite testing, only two (11%) had microsatellite instability-high (MSI-H) tumors. Most patients with available Combined Positive Score (CPS) values had a CPS ≥ 1 (77%). All patients underwent laparoscopic evaluation before treatment initiation and had no evidence of peritoneal malignancy.

### 3.1. Safety

Table 2 summarizes toxicity data.

No grade 5 treatment-related AEs occurred. Grade 4 AEs were observed in three patients (10%), including acute kidney injury [18], neutropenia, and myocarditis/myositis/myasthenia gravis (overlap syndrome) requiring pacemaker placement. This patient’s cardiac function is currently stable as well as his myasthenic symptoms, which are managed with daily pyridostigmine. Two patients (those with overlap syndrome and acute kidney injury) discontinued study treatment to manage these toxicities; both remain alive. Most common grade 3 AEs were nausea (10%, n = 3), fatigue (10%, n = 3) and abdominal pain (7%, n = 2). Among the 13 patients with grade ≥ 3 AEs, only one patient who had acute kidney injury did not proceed to surgery due to poor medical condition. Furthermore, a patient developed grade 2 adrenal insufficiency prior to surgery, resulting in significant surgical challenges; the patient required lifelong steroid replacement and subsequently died of progressive peritoneal disease four months after surgery. A second patient who also had grade 2 adrenal insufficiency prior to surgery remains on lifelong steroid replacement and is alive with no evidence of disease. Neither grade ≥ 3 AEs nor immunotherapy-related AEs were observed past the neoadjuvant setting.

### 3.2. Pathologic Outcomes

Table 3 summarizes surgical pathology results.

Seven patients did not undergo surgery; five of them were due to progression of disease (three of them after chemoradiation) and two due to clinical deterioration.

Among the 23 patients who underwent resection, 9 achieved pCR (39.1%; 95% CI: 19.7–61.5%) and 15 had TNM downstaging. Two patients had MSI-H tumors, one of whom achieved pCR. Among patients with MSS tumors, 2 of 16 achieved pCR. In the intention-to-treat analysis (ITT) population (n = 30), the pCR rate was 30% (95% CI: 14.7–49.4%).

Twelve patients (52.2%; 95% CI: 30.6–73.2%) had ≤1% residual tumor, corresponding to 40% of the ITT cohort (95% CI: 22.7–59.4%). R0 resection was achieved in 20 of 23 surgical patients (87%; 95% CI: 66.4–97.2%) and in 66.7% of the ITT cohort (95% CI: 47.2–82.3%).

### 3.3. Overall Survival

**Figure 2:**
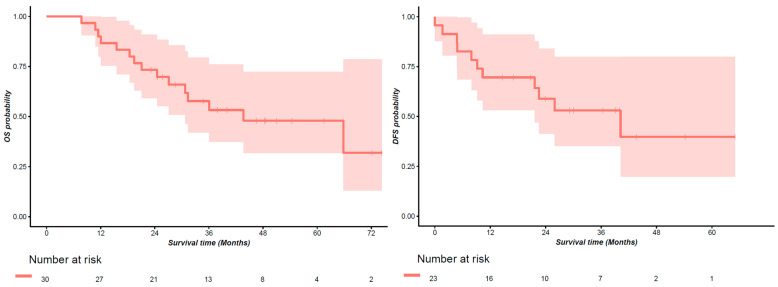
Overall survival and disease-free survival Kaplan–Meier curves. Each vertical line indicate censored patients/last follow-up. Shadows refer to 95% confidence interval.

At the time of analysis, 15 of the 30 patients had died. The estimated median OS (Figure 2) was 43.7 months (95% CI: 30.7–not estimable). Estimated survival probabilities were a 2-year OS 73.3% (95% CI: 0.591–0.91), 3-year OS 57.5% (95% CI: 0.419–0.795), and 5-year OS 47.9% (95% CI: 0.318–0.724). The estimated median follow-up was 48.4 months (95% CI: 40–not estimable).

### 3.4. Disease-Free Survival

DFS was assessed in 23 patients who underwent resection. Eleven patients experienced disease recurrence or death. The estimated median DFS (Figure 2) was 40.2 months (95% CI: 21.6–not estimable). Estimated DFS at 1 and 2 years were 69.6% (95% CI: 0.531–0.912) and 58.9% (95% CI: 0.351–0.8), respectively.

Higher progression/recurrence rates were associated with SRC component (*p* = 0.009) but not with baseline staging and histological grade.

### 3.5. Pathological Response and Survival

**Figure 3:**
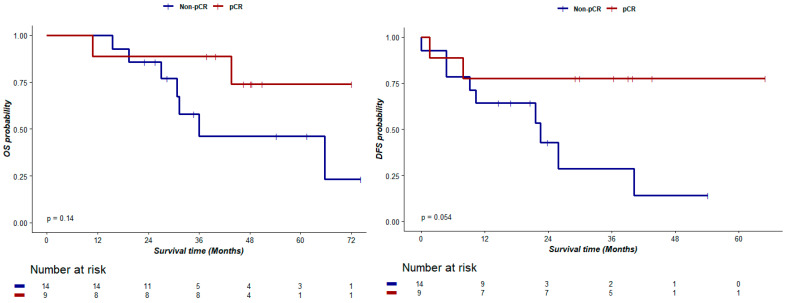
Overall survival and disease-free survival Kaplan–Meier curves by pathological response.

Median OS and DFS were analyzed in the resection cohort to assess differences in survival when pCR is achieved (Figure 3).

Among the nine patients with pCR, there were no disease recurrences but two deaths. Median OS and DFS were not reached, the 2-year OS probability was 88.9% (95% CI: 0.706–1) and the 2-year DFS probability was 77.8% (95% CI: 0.549–1).

Among 14 patients without pCR, there were 2 disease recurrences and 7 deaths. Median OS was 36 months (95% CI: 0.307–not estimable), and the 2-year OS probability was 85.7% (95% CI: 0.692–1). Median DFS was 22.5 months (95% CI: 10.3–not estimable), and the 2-year DFS probability was 42.9% (95% CI: 0.216–0.852).

Although outcomes trended more favorably in the pCR group, the differences were not statistically significant for OS and DFS (log rank test *p* = 0.14 and 0.054, respectively).

### 3.6. Biomarkers

Biomarker correlatives analyses are ongoing. Tissue was collected before and after treatment to support evaluation of the tumor immune microenvironment, immune activation, and stromal remodeling. The results will be reported in a separate manuscript.

## 4. Discussion

To our knowledge, this study is among the first prospective evaluations on safety and pathologic regression outcomes of a perioperative dual IO strategy combined with standard chemotherapy, chemoradiation, and surgery for localized gastric adenocarcinoma. Our study demonstrated encouraging tumor regression and manageable toxicity with incorporation of immunotherapy into a chemoradiation-based perioperative strategy.

Direct comparison with DANTE and METTERHORN should be interpreted cautiously because these studies evaluated perioperative chemotherapy plus immunotherapy without radiation [19,20,21], whereas our study incorporated chemoradiation as a central component of treatment. Consequently, differences in pCR rates likely reflect differences in treatment platforms rather than isolated effects of immunotherapy. These studies nevertheless support the broader movement toward incorporating immunotherapy into perioperative treatment strategies.

The EA2174 trial represents the largest prospective study evaluating incorporation of dual-checkpoint inhibition into a chemoradiation platform for localized gastroesophageal adenocarcinoma. Although pCR rates were higher with nivolumab/ipilimumab plus chemoradiation than with chemoradiation alone, differences were not statistically significant [22]. Nonetheless, EA2174 confirmed the feasibility of incorporating immunotherapy into multimodality treatment and highlighted the need for improved patient selection and optimization of treatment sequencing. A complementary benchmark is CALGB 80803, a randomized phase II PET-directed study in which patients received induction chemotherapy, followed by PET-adapted chemoradiation and surgery. PET responders treated with induction FOLFOX achieved a pCR rate of 40.3% and a median OS of 48.8 months [23]. The pCR rate of 39.1% and median OS of 43.7 months observed in our cohort were similar to outcomes reported in CALGB 80803. This comparison suggests that the pathological response observed in our study cannot be attributed solely to the incorporation of immunotherapy and highlights the complexity of cross-study comparisons.

It is important to note that, in this trial, 23.3% of the patients did not undergo surgery, either due to progression or clinical decline. This rate is comparable to those observed in EA2174 (19%). Patients who failed to undergo surgery were offered the standard of care. Perioperative mortality and morbidity rates among gastric cancer patients undergoing D2 gastrectomy are around 3% and 20%, respectively. Most common complications are respiratory (up to 13%), pancreatic fistula (up to 10%), bleeding (up to 4%) and anastomotic leak (up to 3.5%) [24,25,26]. In this cohort, no adverse events related to surgical procedures were observed. Grade 3 and 4 adverse events were observed in 43.3% of patients, while these rates were 69% in DANTE and 71.6% in MATTERHORN. Adrenal insufficiency and overlap syndrome have been previously reported in nivolumab plus ipilimumab regimens and thus require careful surveillance [27,28,29].

A notable characteristic of our cohort was the high prevalence of diffuse histology (63%) and signet ring cell features (43%), which are generally associated with infiltrative growth patterns and poorer responses to multimodality therapy. Although diffuse tumors may exhibit microscopic extension beyond radiographically apparent disease, recurrence patterns in our study were predominantly distant rather than local (93% versus 7%), suggesting that disease biology rather than inadequate local treatment volume is a major contributor to progression events. Nonetheless, MSI-H and higher PD-L1 CPS expression levels predict IO benefit in gastroesophageal adenocarcinomas, while claudin 18.2 expression may exert some adverse impact [30,31].

As treatment paradigms continue to evolve toward universal incorporation of IO, our results highlight the ongoing relevance of understanding how radiation may synergize with immune checkpoint blockade to enhance local and systemic tumor control. Comparative studies will be needed to clarify how immune-chemoradiation approaches may best integrate with or differ from emerging FLOT-IO standards.

### Limitations

A key limitation of this study is the relatively small sample size, underscoring the need for validation in a larger, multi-center, multi-arm trial. In addition, as biomarker analyses are still ongoing, our ability to assess differences in treatment response and tolerability across molecular subgroups remains limited, but may become more informative as additional data emerge. Safety was a central focus, and while most AEs mirrored those expected with standard therapies, immune-related toxicities such as myocarditis and adrenal insufficiency were observed. These events required long-term medical management and emphasize the importance of continued clinical and laboratory monitoring, not only during neoadjuvant and adjuvant treatment but also throughout surveillance.

## 5. Conclusions

Overall, our neoadjuvant approach incorporating IO plus chemotherapy followed by immune-chemoradiation, surgical resection, and adjuvant immunotherapy demonstrated promising tumor regression and supports the continued investigation of radiation-enhanced immunotherapy for localized gastric and GEJ adenocarcinoma. Longer follow-up is necessary to determine whether the improved pCR rates translate into survival benefit. Ongoing analysis of serial tumor samples may further elucidate how IO, chemotherapy and radiation collectively remodel the tumor immune microenvironment and may guide future personalization of perioperative treatment.

## Figures and Tables

**Table 1:** Baseline clinical characteristics.

Characteristic	n = 30
**Age**, median (min, max)	58 (41, 80)
**Race**	**n (%)**
White	22 (73)
Black or African American	2 (7)
Native American	1 (3)
Asian	1 (3)
Other race	4 (13)
**Ethnicity**	**n (%)**
Not Hispanic or Latino	20 (67)
Hispanic or Latino	10 (33)
**Gender**	**n (%)**
Male	17 (57)
Female	13 (43)
**BMI**, median (min, max)	29 (19, 52)
**ECOG-PS**	**n (%)**
0	16 (53)
1	14 (47)
**Alcohol use**	**n (%)**
Not currently	10 (33)
Never	8 (27)
Frequent	6 (20)
Occasional	6 (20)
**Smoking history**	**n (%)**
Nonsmoker	15 (50)
Nonsmoker-quit	14 (47)
Smoker	1 (3)
**Past or synchronous malignancy**	**n (%)**
None	23 (77)
Prostate cancer	2 (7)
Basal cell carcinoma of skin	1 (3)
Melanoma	1 (3)
Sarcoma	1 (3)
Skin cancer	1 (3)
Thyroid	1 (3)
**History of systemic or radiation therapy**	**n (%)**
None	28 (93)
Chemotherapy	1 (3)
Radiation and hormone ablation for prostate cancer	1 (3)
**Tumor location**	**n (%)**
Antrum	8 (27)
Fundus	7 (23)
Body	6 (20)
GEJ	6 (20)
Cardia	2 (7)
Pylorus	1 (3)
**Site/Siewert classification**	**n (%)**
GEJ Siewert 1	1 (3)
GEJ Siewert 2	5 (17)
GEJ Siewert 3	1 (3)
Gastric	23 (77)
**Baseline T**	**n (%)**
T2	3 (10)
T3	22 (73)
T4	1 (3)
T4A	3 (10)
T4B	1 (3)
**Baseline N**	**n (%)**
N0	13 (43)
N1	13 (43)
N2	3 (10)
N3	1 (3)
**Baseline M**	**n (%)**
M0	30 (100)
**cStage**	**n (%)**
I	2 (7)
IIB	10 (33)
III	17 (57)
IVA	1 (3)
**Adenocarcinoma subtype**	**n (%)**
NOS—not otherwise specified	16 (53)
SRC—signet ring cell	14 (47)
**Tumor grade**	**n (%)**
G3—Poorly differentiated	20 (67)
G2—Moderately differentiated	8 (27)
Moderate to poorly differentiated	2 (7)
**Histological type (Lauren)**	**n (%)**
Diffuse	19 (63)
Intestinal	6 (20)
N/S	5 (17)
**HER2**	**n (%)**
Negative	18 (60)
Equivocal (2+)	1 (3)
N/S	11 (37)
**PD-L1**	**n (%)**
Positive	10 (33)
Negative	3 (10)
N/S	17 (57)
**CPS**	**n (%)**
<1	3 (10)
≥1	10 (33)
N/S	17 (57)
**Microsatellite instability**	**n (%)**
MSS/MSI-L	16 (53)
MSI-H	2 (7)
N/S	12 (40)
***Helicobacter pylori***	**n (%)**
Negative	26 (87)
Positive	1 (3)
N/S	3 (10)

BMI: body mass index; ECOG-PS: Eastern Cooperative Oncology Group performance status; GEJ: gastroesophageal junction; MSI-H: high microsatellite instability; MSI-L: low microsatellite instability; MSS: microsatellite stable; N/S: not specified.

**Table 2:** Serious adverse event summary and adverse events occurring in 10% of patients or more, by grade.

Adverse Event	Grade, No. (%) of Patients		
	1 or 2	3	4
Neutropenia	2 (7)	1 (3)	1 (3)
Acute kidney injury	1 (3)	1 (3)	1 (3)
Overlap syndrome	0	0	1 (3)
Nausea	27 (90)	3 (10)	0
Vomiting	19 (63)	3 (10)	0
Abdominal pain	19 (63)	2 (7)	0
Fatigue	24 (80)	1 (3)	0
Anorexia	21 (70)	1 (3)	0
Anemia	10 (33)	1 (3)	0
Hypokalemia	8 (27)	1 (3)	0
ALT increased	4 (13)	1 (3)	0
Dehydration	4 (13)	1 (3)	0
aPTT increased	1 (3)	1 (3)	0
Hypotension	1 (3)	1 (3)	0
Atrial fibrillation	0	1 (3)	0
Gastritis	0	1 (3)	0
Hypertension	0	1 (3)	0
Hyponatremia	0	1 (3)	0
Syncope	0	1 (3)	0
Diarrhea	24 (80)	0	0
Constipation	23 (77)	0	0
Dysphagia	17 (57)	0	0
Dizziness	13 (43)	0	0
Headache	13 (43)	0	0
Insomnia	13 (43)	0	0
Peripheral sensory neuropathy	13 (43)	0	0
Dyspnea	12 (40)	0	0
Oral mucositis	12 (40)	0	0
Peripheral motor neuropathy	12 (40)	0	0
Cough	11 (37)	0	0
Anxiety	10 (33)	0	0
Gastroesophageal reflux	10 (33)	0	0
Arthralgia	8 (27)	0	0
Blurred vision	8 (27)	0	0
Fever	7 (23)	0	0
Abdominal distension	6 (20)	0	0
Back pain	6 (20)	0	0
Rash acneiform	6 (20)	0	0
Papulopustular rash	4 (13)	0	0
Anal hemorrhage	3 (10)	0	0
Arthritis	3 (10)	0	0
Creatinine increased	3 (10)	0	0
Depression	3 (10)	0	0
Edema limbs	3 (10)	0	0
Hypothyroidism	3 (10)	0	0
Myalgia	3 (10)	0	0
Stomach pain	3 (10)	0	0

ALT: alanine aminotransferase; aPTT: activated partial thromboplastin time.

**Table 3:** Summary of pathological outcomes, response and relapse.

Characteristic		n = 30 (n = 23 Surgery)
Primary Tumor, No. (%)		
	pT0	9 (39)
	pT1a	1 (4)
	pT1b	1 (4)
	pT2	3 (13)
	pT3	6 (26)
	pT4A	3 (13)
Regional LN, No. (%)		
	pN0	11 (48)
	pN1	6 (26)
	pN2	3 (13)
	pN3	2 (9)
	pN3A	1 (4)
Distant Metastasis, No. (%)		
	pM0	22 (96)
	pM1	1 (4)
Surgical Stage, No. (%)		
	0	9 (39)
	I	2 (9)
	II	6 (26)
	III	4 (17)
	IIIB	1 (4)
	IVB	1 (4)
Histologic Grade, No. (%)		
	G2—Moderately Differentiated	3 (21)
	G3—Poorly Differentiated	11 (79)
Pathologic Response, No. (%)		
	P0	9 (39)
	P1	9 (39)
	P2	5 (22)
Residual Cancer, No. (%)		
	0	9 (39)
	<1	2 (9)
	1	1 (4)
	3	1 (4)
	10	1 (4)
	30	1 (4)
	40	1 (4)
	50	1 (4)
	90	1 (4)
	100	3 (13)
	N/S (>0)	2 (9)
Tumor Regression Grade (0–3), No. (%)		
	0	9 (39)
	1	4 (17)
	2	6 (26)
	3	4 (17)
Treatment Response, No. (%)		
	* cCR	1 (3)
	* Non cCR	1 (3)
	Non pCR	14 (47)
	pCR	9 (30)
	Progression—no surgery	5 (17)
Curative or cCR, No. (%)		
	No	11 (38)
	Yes	18 (62)
Residual Disease, No. (%)		
	R0	20 (87)
	R1 (proximal margin)	1 (4)
	R1 (radial margin)	2 (9)
LVI, No. (%)		
	No	17 (74)
	Yes	6 (26)
Positive LN, Median (Min, Max)	n = 23	1 (0, 38)
Total LN, Median (Min, Max)	n = 23	28 (8, 64)
Relapse/Progression, No. (%)		
	No	16 (53)
	Yes	14 (47)
Site of Relapse, No. (%)		
	Distant	14 (93)
	Local	1 (7)
First Site of Relapse, No. (%)		
	Adrenal	1 (7)
	Colon	1 (7)
	Esophageal anastomosis	1 (7)
	Left cervical LN	1 (7)
	Left supraclavicular LN	1 (7)
	Liver	3 (20)
	Pericardial	1 (7)
	Peritoneal	5 (33)
	Subcarinal LN	1 (7)

cCR: clinical complete response; pCR: pathologic complete response; LN: lymph node; LVI: lymphovascular invasion. *: patients who did not undergo surgery due to poor medical condition, precluding pathological response assessment.

## Data Availability

The data generated and analyzed in this study are not publicly available due to patient privacy and institutional regulatory restrictions. De-identified datasets may be made available from the corresponding author upon reasonable request and with appropriate institutional approvals.
